# Brain reorganization as a function of walking experience in 12-month-old infants: implications for the development of manual laterality

**DOI:** 10.3389/fpsyg.2014.00245

**Published:** 2014-03-21

**Authors:** Daniela Corbetta, Denise R. Friedman, Martha Ann Bell

**Affiliations:** ^1^Department of Psychology, University of TennesseeKnoxville, TN, USA; ^2^Department of Psychology, Roanoke CollegeSalem, VA, USA; ^3^Department of Psychology, Virginia TechBlacksburg, VA, USA

**Keywords:** brain reorganization, human infants, reaching, walking, manual laterality, EEG coherence

## Abstract

Hand preference in infancy is marked by many developmental shifts in hand use and arm coupling as infants reach for and manipulate objects. Research has linked these early shifts in hand use to the emergence of fundamental postural–locomotor milestones. Specifically, it was found that bimanual reaching declines when infants learn to sit; increases if infants begin to scoot in a sitting posture; declines when infants begin to crawl on hands and knees; and increases again when infants start walking upright. Why such pattern fluctuations during periods of postural–locomotor learning? One proposed hypothesis is that arm use practiced for the specific purpose of controlling posture and achieving locomotion transfers to reaching via brain functional reorganization. There has been scientific support for functional cortical reorganization and change in neural connectivity in response to motor practice in adults and animals, and as a function of crawling experience in human infants. In this research, we examined whether changes in neural connectivity also occurred as infants coupled their arms when learning to walk and whether such coupling mapped onto reaching laterality. Electroencephalogram (EEG) coherence data were collected from 43 12-month-old infants with varied levels of walking experience. EEG was recorded during quiet, attentive baseline. Walking proficiency was laboratory assessed and reaching responses were captured using small toys presented at mid-line while infants were sitting. Results revealed greater EEG coherence at homologous prefrontal/central scalp locations for the novice walkers compared to the prewalkers or more experienced walkers. In addition, reaching laterality was low in prewalkers and early walkers but high in experienced walkers. These results are consistent with the interpretation that arm coupling practiced during early walking transferred to reaching via brain functional reorganization, leading to the observed developmental changes in manual laterality.

## INTRODUCTION

The development of hand preference in the first year of life has been described by many researchers as an unstable process marked by many shifts in hand use and arm coupling as infants learn to reach for and manipulate objects (Gesell and Ames, 1947; Goldfield and Michel, 1986; Corbetta and Thelen, 1996, 1999; Fagard and Pezé, 1997). Several studies have linked these developmental shifts in early goal-directed hand use to the emergence of fundamental postural and locomotor milestones. For example, Rochat (1992) documented a decline in bimanual reaching and an increase in one-handed reaching when infants learned to sit independently. Goldfield (1993), Corbetta and Thelen (2002), and Babik et al. (2014) further documented such decoupling in hand use in relation to the onset of hands-and-knees crawling. Finally, Corbetta and Bojczyk (2002), and more recently Berger et al. (2011) and Babik et al. (2014) observed a return to two-handed reaching toward the end of the first year when infants learned to stand and performed their first independent steps. This return to two-handed reaching was especially surprising given that the infants in those studies had been followed longitudinally since the age of 6–8 months. They had demonstrated the ability to reach for small objects with one hand for several months prior to walking onset, and as a result of such regular follow-up had become quite familiar with the task and at practicing one-handed reaching. This increase in bimanual reaching at the onset of upright locomotion was also found to be accompanied with a decline in preferred hand use (Corbetta and Thelen, 2002; Berger et al., 2011; although see Babik et al., 2014).

In most of these studies, the observed developmental fluctuations in bimanual reaching were not directly associated with the act of locomoting *per se* – fluctuations in arm use were documented when infants were sitting while reaching. Yet, the fact that changes in patterns of hand use in reaching occurred during specific periods of whole body postural reorganizations and gross motor skills learning suggested that some underlying developmental process might have linked changes in reaching with the learning of the new fundamental motor skills being acquired. Following this reasoning, Corbetta and Bojczyk (2002) proposed a *transfer of learning* account (see also Corbetta and Thelen, 2002; Corbetta et al., 2006; Corbetta, 2009). In the context of self-produced locomotion, they argued that the novel and specific arm use activity associated with the processes of maintaining balance and coordinating arms and body movements to propel the body forward might have temporarily transferred to reaching until these gross motor skills were acquired, or became more routine-like. Specifically, the break in reaching coupling associated with the emergence of hands-and-knees crawling was seen as the product of actively learning to sequence and alternate the movements of the forearms in order to crawl. This act of newly practicing arm alternation during self-produced quadruped locomotion, in turn was assumed to have transferred to reaching, hence enticing the shift to a greater use of alternated, one-handed, non-lateralized reaching responses during that period of development (Corbetta and Thelen, 1999, 2002). Likewise, the return to bimanual reaching and continued decline in lateralized hand use observed toward the end of the first year was seen as the product of the extensive upper arm coupling that infants produce when actively controlling their upright balance with their arms in high guard position; i.e., with arms held up at or above shoulder level during stepping. Such arm coupling during early walking was considered to have transferred to reaching, hence again, entraining the rise in bimanual reaching responses documented during this critical learning period of upright balance control (Corbetta and Bojczyk, 2002; Corbetta and Thelen, 2002; Corbetta et al., 2006).

The pattern resemblance observed between the transient responses adopted in reaching and the specific arm use being practiced during specific postural–locomotor skill learning is consistent with the hypothesis that some transfer of learning may have occurred between locomotor and reaching skills; however, why and how such transfer would occur remains unclear. Corbetta and Bojczyk (2002) speculated that such transfers in behavioral patterning between locomotion and reaching might have occurred via functional brain reorganization. To support their arguments, these researchers referred to a number of classic studies in the neurosciences. These studies, performed with adults and animals, have demonstrated the effects of specific motor practice and novel sensory-motor experiences on brain plasticity and cortical functional reorganization, particularly in the sensory-motor cortex (Jenkins et al., 1990; Merzenich and Jenkins, 1993; Karni et al., 1998; Kleim et al., 1998; Petersen et al., 1998, to cite a few). Of particular interest is the fact that these studies found cortical reorganization to be closely related to the task that was being learned and practiced, and hence, to the specific limbs, body parts, and sensory organs that were used to achieve the task. Some studies even found a direct mapping between brain hemispheric organization and upper arm coupling. For example, Andres et al. (1999) have shown that the acquisition of bimanual skills increased coupling of homologous cortical sensory-motor areas. And bilateral versus unilateral limb training in a reaching task was found to differentially affect dendritic branching of neurons in the rat motor-sensory forelimb cortex (Greenough et al., 1985). Likewise, the use of one arm more than the other was linked to a larger upper limb representation in the hemisphere contralateral to the hand mostly used (Nudo et al., 1996). Such brain and behavior mapping was shown to be powerful for motor function rehabilitation in stroke patients who lost the use of one arm; the intensive coupled training of the activity of both arms helped function recovery of the hemiparetic arm (Luft et al., 2004; Waller and Whitall, 2005). Together, these studies stress how specific motor activity can drive neuromotor reorganization. But this process of reorganization can go both ways. A study found that practice-dependent neural reorganization can, in turn, shape motor performance (Dorris et al., 2000). Thus, as a whole, these studies point to the constant mapping existing between brain and behavior as new sensory-motor skills are being learned, practiced, and assimilated.

Corbetta and Bojczyk (2002) suspected that a similar kind of mapping could have occurred between the emergence of novel forms of locomotion, reaching patterning, and the brain. When infants learn new fundamental motor skills such as crawling or walking, they need to learn how to use their body in a new way (Adolph et al., 1998; Kubo and Ulrich, 2006; Snapp-Childs and Corbetta, 2009). This involves coordinating and sequencing complex sets of muscles in a manner they never performed before. We also know that when infants discover how to use their body to achieve these new skills, they tend to practice them a lot (Adolph et al., 2012). Corbetta and Bojczyk (2002) reasoned that it was the specific and extensive practice of novel arm activity used in the context of learning these new locomotor skills (i.e., to control balance or generate new limb sequences) that temporarily transferred to reaching via brain functional reorganization. During such critical periods of motor skill learning, the brain is attempting the difficult task of integrating novel and complex forms of movement coordination into the existing motor repertoire of the child. It is possible that this type of sensory-motor integration process is initially achieved by temporarily mapping a novel functional use of some sets of muscles and limbs in some tasks (i.e., locomotion) with prior existing functional uses of these same sets of muscles and limbs in other tasks (i.e., reaching). Such mapping could lead to a period of temporary, undifferentiated responses across tasks in the process of integrating the new skill in the existing motor repertoire of the child. Differentiation between skills would progressively take place as mastery and control of the new emerging skills would form. In walking, for example, the upper arm coupling adopted during the first weeks of upright locomotion (Ledebt, 2001; Corbetta and Bojczyk, 2002) may become a preferred mode of arm use for children, due to their poor upright balance control and extensive practice at coupling their arms during stepping (Kubo and Ulrich, 2006). This increase in upper arm coupling may be mapped onto the corresponding cortical sensory-motor areas controlling the upper arms, thus driving an increased cortical representation of arm coupling during this period of learning. In turn, this representation could be transferred or mapped onto the same set of muscles during reaching, even though infants are not walking, but sitting while reaching, and have reached using different patterns prior to the onset of upright locomotion. Thus, combining identical groups of muscles in similar functional ways across tasks and behaviors could be a temporary solution to facilitate the sensory-motor integration of the new motor skill into the existing motor repertoire of the child. Consistent with such interpretation, Corbetta and Bojczyk (2002) observed that infants maintained coupling in seated reaching as long as they were coupling their arms following the onset of independent walking. When infants improved upright balance control, lowered their arms, and decoupled them, coupling in reaching declined as well.

The same scenario could be applied to the transition to hands-and-knees crawling. When infants are learning to crawl on hands and knees, they figure out how to sequence and alternate arms and legs in order to move their body forward. As they do so, both arms acquire the new role of supporting the body. In that role also, both arms become equally preferred, but in an alternate way, since they are both used sequentially to move the body forward. Thus, as infants learn to crawl on hands and knees, the activities of the arms remain uncoupled but are used in alternation. Consistent with such scenario, Corbetta and Thelen (1999, 2002) found that such uncoupled, alternated, and distributed preferred hand use became the more predominant mode of response in reaching when infants began to practice hands-and-knees crawling. Thus again, the similarity and consistency of patterns of arm use across crawling and reaching could be the result of a temporary, undifferentiated mapping between the brain functionally reorganizing to assimilate the new locomotor skill while maintaining reaching, especially given that both tasks require the use of similar upper arm sets of muscles.

This interpretation that newly practiced patterns of hand use following the emergence of novel forms of locomotion can transfer to reaching was further generalized to other skills through the longitudinal study of two young infants that adopted less common forms of self-produced locomotion (Corbetta et al., 2006). One child, who began to locomote by scooting on his buttocks while in a sitting posture, also began to couple his arms during reaching over the same developmental period. As in prior reports, the rise in reaching coupling that occurred following the emergence of scooting was interpreted as a result of the emergent upper arm coupling that was extensively performed during scooting. Another infant, who, in contrast, preferred to crawl on his belly by dragging his body on the floor by using the same steady, lateralized pattern between hands and legs continued to maintain a strong right hand use for reaching. Unlike other infants who alternated arms for crawling on hands and knees and displayed a disappearance in hand preference, this infant maintained a strong right bias in reaching, presumably as the result of never alternating arm movements during belly crawling. Thus, these two case studies not only confirmed that hand patterns during reaching can reciprocate arm patterns used during specific learning of forms of locomotion but also showed that mapping between arm use during locomotor and reaching tasks can generalize across multiple and varied forms of locomotion.

The goal of this study was to test our hypothesis that functional brain reorganization may underlie the above documented changes and transfer in hand use across locomotor skill learning and goal-directed reaching. We mentioned above that the neuroscience literature offers supportive evidence for such activity-dependent cortical reorganization, but these studies were performed with human adults or animals. Evidence from the human infant literature revealing the occurrence of such activity-dependent brain reorganizations is quite sparse. To our knowledge, the only study that supports such experience-dependent cortical reorganization in early normal development was performed by Bell and Fox (1996) in which they documented changes in Electroencephalogram (EEG) coherence in four groups of 8-month-old infants that differed in their levels of crawling experience. EEG coherence is the frequency-dependent, squared cross-correlation of electrical signals between two scalp electrode sites (Nunez, 1981; Thatcher et al., 1986). Coherence values range from 0 to 1 and Thatcher proposed that coherence indicates the strength and number of synaptic connections (Thatcher, 1994) and, thus, is reflective of the level of connectivity between two cortical sites. High coherence values indicate that cortical regions are intricately linked and working together. Greater connectivity during development, however, does not always indicate greater maturity. At an early period in development, high coherence values may indicate that two distant cortical regions are intricately linked and working together. With maturation, there may be increased regional differentiation and a decrease in coherence. Thus, measures of EEG coherence can be used to investigate early developmental changes in cortical organization or structural connectivity (Bell, 2001, 2012). Furthermore, EEG coherence has been successfully employed by researchers to capture change in brain connectivity between electrode sites as a function of change in coupling between effectors during the motor learning of bimanual tasks in adults and children (e.g., Andres et al., 1999; Serrien and Brown, 2003; de Castelnau et al., 2008).

Bell and Fox (1996) reported an inverted U-shaped function in EEG coherence as a function of increasing crawling experience. Based on resting baseline measures of brain electrical activity, the novice crawlers (with 1–8 weeks experience) displayed greater EEG coherence than either the pre-crawling group or the experienced crawlers. Particularly, changes in EEG coherence were found over the medial frontal/lateral frontal and medial frontal/occipital regions. Bell and Fox interpreted the increase in EEG coherence in novice crawlers as reflecting an increase in synaptic connections between brain sites associated with onset and early experience at crawling. They considered the decrease in EEG coherence in the most experienced crawlers as reflecting a pruning of the overabundant synaptic connections when crawling became more skilled.

Our study aimed to extend the work of Bell and Fox (1996) by examining whether similar changes in EEG coherence could be captured during the transition to upright locomotion in 12-month-old infants, and examine if these changes mapped onto changes in reaching. As in Bell and Fox (1996), we used groups of infants that were age matched but had distinct levels of walking experience (non-walkers, novice walkers, and more experienced walkers) and, as in Corbetta and Bojczyk (2002), we examined these infants’ reaching skills while they were supported in a sitting posture and reaching for small objects presented at midline. With the goal of addressing the issue of transfer of learning discussed above, we predicted, based on Bell and Fox (1996), that novice walkers who are coupling their arms during walking would display increased EEG coherence in resting baseline brain electrical activity relative to prewalking infants. We expected that cortical regions in support of gross motor behaviors related to walking would be linked and working together in the early performance of this newly acquired skill. We also predicted that such increased arm coupling during walking in novice walkers should also occur in reaching while seated, and should result in a lower manual laterality index during reaching (Corbetta and Bojczyk, 2002; Corbetta and Thelen, 2002; Berger et al., 2011). We also hypothesized that as infants acquired experience with walking and decoupled their arms during walking, EEG coherence would decrease as overabundant synapses would be pruned due to increased regional differentiation. Coupling in reaching would also decline, and as a result of arm decoupling, manual laterality would increase.

Finally, because the cortical reorganization we aimed to examine is in relation to increased upper arm coupling of homologous muscles in novice walkers, we predicted that increased EEG coherence should occur in homologous sites of the brain hemispheres. We also had hypotheses about specific brain areas. The motor cortex of the frontal lobes is involved in the planning and execution of movement, such as walking and reaching, but more anterior frontal areas are associated with reaching as well. Using near-infrared spectroscopy with adults, Goto et al. (2011) reported that the lateral prefrontal cortex was involved in reaching that was both perceptually consistent and perceptually effortful. Wallis et al. (2001) demonstrated that monkeys with lesions to the lateral prefrontal cortex had difficulty transferring reaching strategy to a new context. Finally, using EEG, Cochin et al. (1999) reported mu rhythm synchronization at lateral frontal and motor cortex electrode sites, along with some temporal and parietal locations, during observation as well as execution of finger movements. Using the classic 10/20 system of electrode classification, the lateral frontal electrode locations are F7, F8 and the motor cortex locations are (central) C3, C4. Thus, we specifically examined changes in EEG coherence during resting baseline in homologous lateral frontal and motor cortex (F7/C3, F8/C4). Because of the linkages between changing reaching patterns with onset of walking, we hypothesized that novice walkers would show increased frontal/central coherence during resting baseline compared to pre-walking or experienced walkers of the same age.

## MATERIALS AND METHODS

### PARTICIPANTS

Participants were 50 healthy, 12-month-old infants (26 boys and 24 girls) who were recruited from birth announcements placed in the local newspaper. Approximately half of the infants were also participating in a longitudinal study of individual differences in cognitive development and had been in the research laboratory at 5 and 10 months of age for that study (e.g., Diaz and Bell, 2011; Cuevas and Bell, 2013; Kraybill and Bell, 2013). Infants were 96% Caucasian and all parents had a minimum of a high-school diploma. Infants were born within three weeks of their expected due dates and were seen within three weeks following their 12-month birthday, with the exception of one infant who was seen within four weeks. Infants were given a t-shirt or a book for their participation in the study. This study was approved by the Virginia Tech Institutional Review Board.

### PROCEDURES

Upon arrival at the research laboratory, parents were shown the electrophysiological equipment and all research procedures were explained. After obtaining written parental consent, EEG electrodes were applied and the different tasks were performed in the following order: first, a 1-min baseline physiology was recorded while the infant was sitting on the mother’s lap, then reaching while sitting was assessed (the electrodes remained on the scalp during the reaching task), and finally, after removing the EEG cap, infants were encouraged to walk along a corridor to assess their level of self-produced locomotor experience. This task order was chosen and maintained to control for potential lingering effects of arm coupling in reaching and/or walking on EEG coherence and arm coupling of walking on reaching.

#### EEG recording

EEG recordings were accomplished during baseline and during a reaching task. We focus on the baseline EEG data in this report. Recordings were made from frontal pole (Fp1, Fp2), medial frontal (F3, F4), lateral frontal (F7, F8), central (C3, C4), parietal (P3, P4), and occipital (O1,O2) scalp locations. All electrode sites were referenced to Cz during recording. Baseline EEG was recorded for 1 min while the infant sat on the mother’s lap. During the baseline recording, a research assistant blew on a toy pinwheel to make it spin, 1.1 m in front of the infant. This procedure quieted the infant and yielded minimal eye movements and gross motor movements, thus allowing the infant to tolerate the EEG cap for the recording. Mothers were instructed not to talk to their infants during the EEG recording. Immediately after baseline, the reaching task was administered.

EEG was recorded using a stretch cap (Electro-Cap, Inc., Eaton, OH, USA) with electrodes in the 10/20 system pattern. After the cap was placed on the infant’s head, recommended procedures regarding EEG data collection with infants and young children were followed (Pivik et al., 1993). Specifically, a small amount of abrasive gel was placed into each recording site and the scalp was gently rubbed. Following this, conductive gel was placed in each site. Electrode impedances were measured and accepted if they were below 10 K ohms.

The electrical activity from each lead was amplified using separate SA Instrumentation Bioamps (San Diego, CA, USA) and bandpassed from 0.1 to 100 Hz. Activity for each lead was displayed on the monitor of an acquisition computer. The EEG signal was digitized on line at 512 samples per second for each channel so that the data were not affected by aliasing. The acquisition software was Snapshot-Snapstream (HEM Data Corp., Southfield, MI, USA) and the raw data were stored for later analyses. Prior to the recording of each subject a 10 Hz, 50 μV peak-to-peak sine wave was input through each amplifier. This calibration signal was digitized for 30 s and stored for subsequent analysis.

Spectral analysis of the calibration signal and computation of power at the 9–11 Hz frequency band was accomplished. The power figures were used to calibrate the power derived from the subsequent spectral analysis of the EEG. EEG data were examined and analyzed using the EEG Analysis System software developed by James Long Company (Caroga Lake, NY, USA). First, the data were re-referenced via software to an average reference configuration (Lehmann, 1987). The average reference configuration requires that a sufficient number of electrodes be sampled and that these electrodes be evenly distributed across the scalp. Luck (2005) has demonstrated with event-related potential recordings that voltage can be affected by average reference montage when only mid-line electrodes, as opposed to an entire scalp of electrodes, are used. Currently, there is no agreement concerning the appropriate number of electrodes (Davidson et al., 2000; Hagemann et al., 2001; Luck, 2005). Average referencing is considered the optimal configuration when computing coherence between spatially distinct electrodes (Fein et al., 1988). Then, average reference EEG data were artifact scored for eye movements using a peak-to-peak criterion of 100 μV or greater. Artifact associated with gross motor movements over 200 μV peak to peak was also scored. These artifact-scored epochs were eliminated from all subsequent analyses. The data then were analyzed with a discrete Fourier transform (DFT) using a Hanning window of one-second width and 50% overlap. Power was computed for the 6–9 Hz frequency band because infants have a dominant frequency between 6 and 9 Hz (Bell and Fox, 1992; Marshall et al., 2002). Coherence between electrode sites within each hemisphere was computed using an algorithm by Saltzberg et al. (1986). Coherence calculations were performed by averaging the normalized complex cross-spectral density within the 6–9 Hz frequency band across the baseline recording period. Each individual frequency was uniformly weighted within 6–9 Hz band (Saltzberg et al., 1986, Eq. 9). Based on the literature, we focused on EEG coherence between lateral frontal and central scalp locations in both hemispheres (F7/C3, F8/C4).

#### Reaching task

Immediately after baseline physiology recording, the reaching task was administered (Corbetta and Bojczyk, 2002). An experimenter sitting in front of the child removed a toy from under a cover and presented it to the child while saying, e.g., “Look! It’s a frog. Do you want it?” The toy was held for a few seconds out of the infant reach, at infant’s shoulder level, and then moved forward in a straight horizontal path to the infant reaching space while saying “Here, it comes!” followed by the infant reaching. Once the infant had grasped the object, she was given time to explore the object, then, the object was taken away, hidden under the cover, and a new trial began with a new toy. From toy presentation to toy removal, a trial lasted typically about 30 s. Objects for reaching were small toys (balls, animals, rattles, 4–5cm diameter) that infants could easily grasp with one hand. They were presented one at a time, at mid-line, and at infants’ shoulder height. Ten to 11 trials were collected, then the EEG electrode cap was removed and the infant was accompanied in a corridor adjacent to the EEG testing room for walking assessment (see below). The reaching session was videotaped for further behavioral analyses using one single video camera, located at 45° angle on the front left side of the child, allowing visibility of both reaching hand.

Reaching responses were coded from the videos as right, left, or bimanual depending on the arm (R or L) and number of arms (1 or 2) that were extended toward the object during reaching (Corbetta and Thelen, 1996). Right and left codes were used for unimanual arm responses when only one arm (the right or the left) was used to reach for the object. For this code, the non-reaching contralateral arm was not active during the reaching action of the other arm and most commonly remained on the side of the infant body. The bimanual code was used to capture reaching responses in which both arms were coupled in their extension toward the object. Timing between the onset/offset of the arm movements could vary, but movement extensions of both arms toward the target had to overlap during most of the transport duration of the hands toward the target to be coded as bimanual. If one arm reached first, and the second arm began to reach immediately after the first hand had already contacted the target (as in alternated patterns), this response was coded as unimanual, as it did not reflect spatio-temporal coupling between arm movements. Inter-rater reliability for reaching coding was 96.84%. From these data, we computed two variables per infant: (1) a percentage of bimanual responses, which was the number of bimanual reaches divided by the total number of reaching trials performed, and (2) and index of manual laterality was computed using the following equation: $\left(\frac{(R+\left(B/2\right))-\left(L+\left(B/2\right)\right)}{R+L+B}\right),$ where bimanual reaches were split between arms (see Corbetta and Bojczyk, 2002; Jacobsohn et al., 2014).

#### Locomotor assessment

For the last part of the testing, infants were engaged in play activities in a 6-m-long corridor aimed at enticing and capturing their locomotor skills. The mother placed her infant at one end of the corridor, then walked to the other end of the corridor, and encouraged her infant to come. Mothers endorsed that the infants’ choice of locomotion toward them (walking, crawling) was the child’s preferred mode of locomotion. As with the rest of the laboratory visit, the locomotor session was videotaped for further behavioral analyses. Based on the filmed locomotor assessment, infants were assigned to one of the three groups depending on their walking skills and arm coupling during walking: not walking yet (*n* = 18, age average = 12.10 (months/days), SD = 0.013), novice walkers (*n* = 17, age average = 12.05 (months/days), SD = 0.044), and more experienced walkers (*n* = 8, age average 12.09 (months/days), SD = 0.019). Definition of the novice walkers and experienced walkers categories was based on the arm position infants used during walking (as in Corbetta and Bojczyk, 2002). Novice walkers were those infants walking with their arms coupled in high guard position, i.e., above infant waist level. The experienced walkers were those infants walking with their arms decoupled at or below waist level. Reliability coding of walking level and arms position during walking performed on 39% of the infant sample yielded a 100% agreement. Statistical testing confirmed that there were no significant age difference between groups after performing the above classification based on walking experience [Kruskal–Wallis χ ^2^(2) = 1.070, *p* > 0.586].

#### Complete data for analyses

EEG data were available for 43 of the 50 infants (23 boys and 20 girls). Data were lost for seven infants: one due to bioamp failure, one due to extraneous electrical interference in the EEG signal, two due to heart rate interference in the EEG signal, two due to excessive fussiness/crying, and one because both raw and transformed power values were more than 3 SD below the mean of this group of infants. Also, of the 50 infants, 47 of them provided reaching data. One infant refused to reach for the objects and there were technical difficulties with the video recording for two infants. Locomotor assessment was obtained for all 50 infants.

## RESULTS

### BASELINE EEG COHERENCE

**FIGURE 1:**
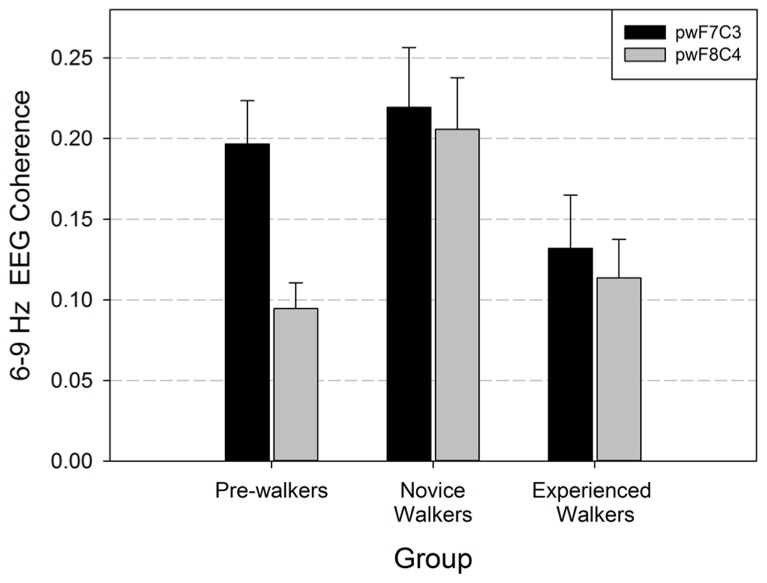
**Lateral frontal/central coherence (F7/C3 = left hemisphere; F8/C4 = right hemisphere) with standard errors during baseline (pinwheels or pw) recording for the three locomotor groups**. Data are graphed from the perspective of the between subjects factor (walk group).

We performed repeated-measures MANOVA on the EEG coherence data recorded during baseline. Based on our hypotheses, we focused on the homologous lateral frontal/central coherence pairs (F7/C3, F8/C4). Hemisphere was the within-subjects factor and walking group was the between-subjects factor. There was a main effect for walking group, *F*(1,40) = 3.367, *p* = 0.045, and a main effect of hemisphere, *Wilks *= 0.906, approximate *F*(1,40) = 4.172, *p* = 0.048. The group by hemisphere interaction was not significant (*p* = 0.124). *Post hoc* analyses were done to determine which groups differed with respect to frontal/central coherence. As seen in **Figure 1**, non-walkers and novice walkers differed in EEG coherence (*p *= 0.001), novice and experienced walkers differed (*p* = 0.005); however, there was no difference in EEG coherence values between the non-walker and experienced walker groups (*p* = 0.246). Thus, the novice walkers had the greatest EEG coherence values and this change occurred on both homologous sites.

To assess whether changes in EEG coherence as a function of walking experience were solely limited to the predicted lateral frontal/central pairs (F7/C3, F8/C4), we ran additional hemisphere × group repeated-measures MANOVAs on all other electrode pair combinations. These analyses revealed no other significant changes in EEG coherence as a function of walking experience (all *p’* s > 0.07).

We also examined whether these walking group differences in EEG coherence were accompanied by group differences in EEG spectral power. MANOVA analysis of the electrodes of interest (F7, F8, C3, C4) revealed no main effects or interactions with walking group (all *p’* s > 0.70). This suggests that cortical activation at medial frontal and central scalp locations was similar for the three walking groups.

### COUPLING AND LATERALITY IN REACHING

**FIGURE 2:**
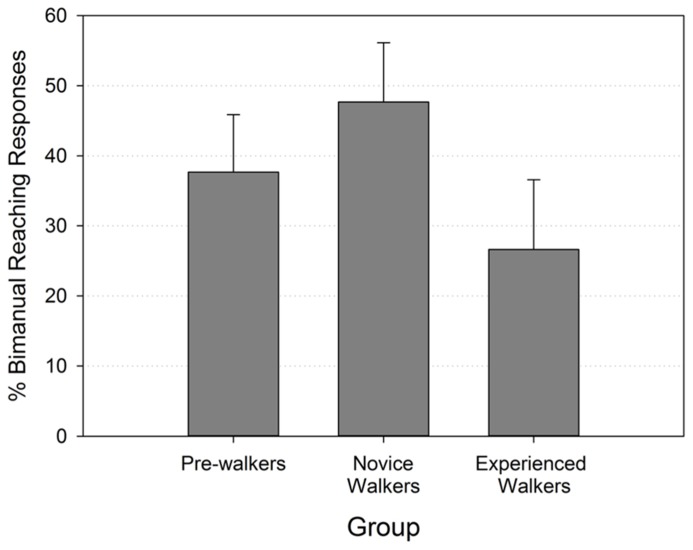
**Mean percentages of bimanual reaching responses (with standard errors) by walking group**.

A Kruskal–Wallis test performed on the percent of bimanual reaching responses of the three walking groups revealed no significant group differences (*p* > 0.471). **Figure 2** shows that the rate of bimanual reaching followed the predicted trend of a higher value in the novice walker group, but even pairwise comparisons testing did not reveal significant differences between groups (all Mann–Whitney *p*’s > 0.175).

**FIGURE 3:**
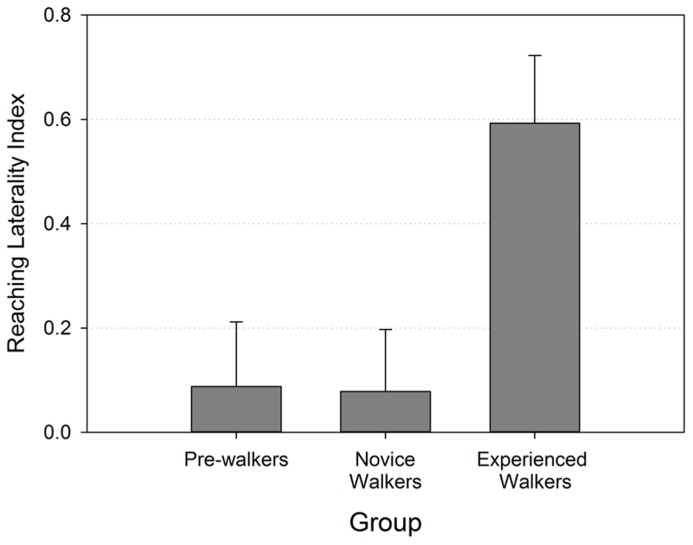
**Reaching laterality index (with standard errors) by walking group**.

The laterality index, on the contrary, revealed strong group differences that were consistent with our predictions. **Figure 3** shows that reaching laterality (specifically right hand use) was significantly greater for the experienced walker groups compared to the two other groups (Kruskal–Wallis χ ^2^ (2) = 6.587, *p* = 0.037). Follow-up pairwise group comparisons confirmed that the experienced walkers used their right hand for reaching significantly more than both the novice walkers [Mann–Whiney *U* = 32.50, *p* = 0.015 (two-tailed)] and non-walkers [Mann–Whiney *U* = 33.00, *p* = 0.022 (two-tailed)]. The non-walkers and novice walkers did not differ from each other in their preferred hand use (*p* > 0.966) and both revealed laterality indexes that were close to 0.

## DISCUSSION

The goal of this research was threefold: first, we wanted to assess whether novice walkers who are coupling their arms in the early stages of learning to walk would display increased structural EEG coherence in resting baseline brain electrical activity relative to same age prewalking infants and more experienced walkers who are not coupling their arms as much for self-produced locomotion. Second, we wanted to assess whether differences in structural EEG coherence would occur bilaterally in homologous brain sites and specifically in brain areas that have been associated with motor planning and motor execution [i.e., motor areas (C3/C4) and lateral frontal areas (F7/F8)]. And third, we wanted to assess whether different levels of walking experience and their associated levels of EEG coherence would also map onto infants’ arm coupling and manual laterality during seated reaching. This last goal was aimed at assessing our hypothesis on transfer of learning from walking to reaching in relation to the increase in bimanual coupling and decrease in manual laterality documented by prior studies at the time infants are learning to walk (Corbetta and Bojczyk, 2002; Berger et al., 2011; Babik et al., 2014).

The majority of our results were consistent with the predictions we made in relation to those three goals. First, we observed an increase between pre- and novice walkers and a decrease between novice and more experienced walkers in structural resting EEG coherence. Recall that all infants in the study were the same age to minimize age confound in our data and to focus more readily on differences in self-produced locomotor experience between groups. This EEG coherence pattern across groups replicated and extended prior findings from Bell and Fox (1996) who reported a similar, inverted U-shaped pattern in EEG coherence in relation to the infants’ crawling experience. Thus, these data show that the emergence of novel and distinct gross motor milestones occurring at different periods of early development are repeatedly associated with patterns of cortical reorganizations and changes in brain connectivity. In the Bell and Fox (1996) and this study, increase in EEG coherence (and assumed increase in synaptic connectivity across brain sites) was found in the novice motor learners, during periods of critical skill development, but not in the premotor learner and the more experienced motor performers.

It should be noted that the coherence findings in our study could be affected by volume conduction (i.e., cortical activity recorded at one scalp location contributing to the signal at other nearby scalp locations). There is evidence, however, that volume conduction effects are much smaller in infants than adults because of their thinner skulls (Grieve et al., 2003). Furthermore, as there were no differences in EEG spectral power across walking groups, it is unclear whether volume conduction differences across groups would have affected the findings.

Second, in this study and as we predicted, the observed change in EEG coherence associated with early walking occurred across homologous motor and lateral frontal brain sites in relation to arm coupling during walking. Recall that the pre, novice, and more experienced walker groups were defined as a function of the infant arm coupling observed during walking. This particular finding met our expectations of how, where in the brain, and in which group increase in EEG coherence should have occurred, and is consistent with our hypothesis that such bilateral structural cortical reorganization may reflect the greater arm coupling practiced during the initial period following the emergence of independent, bipedal walking.

The last results only partially met our predictions. They concerned the coupling and laterality patterns of goal-directed reaching that were actually produced by the infants. According to our hypothesis on transfer of learning, and in line with prior findings from longitudinal studies (Corbetta and Bojczyk, 2002; Berger et al., 2011; Babik et al., 2014), we also expected greater bimanual reaching in the novice walker group compared to the two other infant groups. Furthermore, we expected lower manual laterality in the prewalker and novice walker groups compared to the more experienced walkers. As discussed in the section “Introduction”, prewalking infants are more likely to alternate arm use during hands-and-knees crawling, and novice walkers are more likely to couple their arms during walking, both of which were found to correspond to lower levels of lateralized hand use in reaching compared to more experienced walkers (Corbetta and Thelen, 2002; Berger et al., 2011). We found a weak, non-significant increase in arm coupling in the novice walker group but verified the predicted increase in manual laterality trends as a function of the locomotor experience groups. We think that this partial support of our transfer of learning hypothesis can easily be explained by the cross-sectional study design that we adopted for the purpose of this study, which was aimed at comparing EEG coherence in same-aged infants, albeit with different skills.

All the studies that reported a link between changes in arm use in reaching as a function of emerging locomotor skills used longitudinal designs (Corbetta and Bojczyk, 2002; Corbetta and Thelen, 2002; Corbetta et al., 2006; Berger et al., 2011; Babik et al., 2014). Longitudinal designs allowed researchers to identify change in behavior over time more accurately despite wide individual differences in skill onsets and notable variations in developmental trajectories (see Corbetta and Bojczyk, 2002; Berger et al., 2011; Jacobsohn et al., 2014). What makes increases (or declines) in bimanual reaching following the onsets of locomotor skills particularly identifiable is the change in baseline behavior over several weeks in a row. These changes in baseline behavior can be identified despite the week-to-week or day-to-day fluctuations that are typical of infant behaviors. Thus, the observed increase in arm coupling, or decline in manual preference, as those reported by prior studies, are compound results obtained over several weeks of behavioral observation and therefore are more likely to demonstrate more consistent trends across infants over the observed period. Thus, longitudinal designs allowed researchers to capture the regularities across infant behavioral changes more reliably despite the high response variability intrinsic to infant behavior.

These advantages are not present when behaviors are observed over one single session, as in this study. Single-session observations are more likely to be subject to data inconsistencies due to fluctuation in behavior over time and time of sampling. Prior longitudinal studies that displayed changes in bimanual and lateral reaching in infants over extended periods of time have shown how unstable and fluctuating the week to week infant reaching patterns can be, despite periods of identifiable behavioral trends (see Corbetta and Thelen, 1996, 1999; Corbetta and Bojczyk, 2002). Thus, depending on the day the data were collected, results may not always reflect the overall trend of increased arm coupling in reaching that would be observed if the behavior were observed over several weeks in a row following the onset of the transition skill.

Measuring arm coupling in infant reaching is another source of data variability. Movement lag variability in infant bimanual reaching can be quite significant, even during periods of predominant bimanual reaching (see Corbetta and Thelen, 1996). Finally, a couple of recent studies have suggested that increased coupling in infant reaching may actually begin to occur in some infants before the onset of upright locomotion. Thurman et al. (2012) observed that the timing of the increase in bimanual reaching in six infants followed longitudinally was more in in line with the onset of standing alone than walking *per se*. And, Atun-Einy et al. (2014) found that increase in bimanual reaching began to show a small rise when infants began cruising. Thus, there may be several reasons for our lack of finding of the predicted significant increase in bimanual reaching in the novice walker group. Namely, the high intra- and interindividual response variability inherent to infant reaching can more readily affect data collected over a single day. And the fact that bimanual reaching may already occur in prewalking infants when standing and cruising could contribute to reducing the expected significant increase in reaching coupling from pre- to novice walkers.

### IMPLICATIONS FOR THE DEVELOPMENT OF MANUAL LATERALITY

Overall, findings from this study continue to counter the view that the development of hand preference in infancy follows a set or a steady developmental progression over time. Rather, data from this research continue to support a more plastic, more malleable view of the development of early hand preference, a view involving a process of complex interactions and integration between multiple developmental systems (i.e., whole body gross motor reorganization, structural and functional cortical reorganization, and goal-directed reaching; Corbetta et al., 2006; Corbetta, 2009). This research is also novel in assessing and linking specific aspects of behavioral learning (i.e., locomotion) and the concomitant behavioral reorganization of prior existing skills (i.e., reaching) with predicted changes in the brain. We think that these documented changes in both behavioral and electrophysiological levels as a function of walking experience are important for our understanding of the development of infant manual laterality.

There is some consensus in the field of developmental laterality that manual preference is not clearly established until the age of 2 or 3 years olds (e.g., McManus et al., 1988; although see Michel, 1981). We have argued in previous work that one reason why infants display highly fluctuating patterns of hand preference in the first years of life is related to the multiple and successive postural reorganizations that infants incur and need to acquire on their way to mastering the upright bipedal locomotion (Corbetta and Thelen, 2002; Corbetta, 2005; Corbetta et al., 2006). Since upright locomotion marks the last step of several gross motor reorganizations in early development, growing and more stable trends in hand use preference should begin to appear after the skill of walking has become more stable and more routine. Furthermore, from this time, infants arms are free from their gross locomotor supporting role (as in crawling), or balance control role (as in sitting or walking), which in turn should contribute to the development of more specific and more differentiated arm and hand use to achieve a greater variety of tasks. We found such increase in manual laterality following the onset of upright locomotion in a longitudinal study (Corbetta and Thelen, 2002). And there are several data from the developmental literature showing a steadier growing of manual laterality in the second year of life as infants engage in more dexterous manual tasks (i.e., Fagard and Marks, 2000; Jacobsohn et al., 2014) and tool use (Kahrs et al., 2013; Rat-Fischer et al., 2013). Increased manual laterality in relation to adopting an upright posture or bipedality has also been found in non-human primates and other mammals that typically do not display preferred hand use at the population level (Giljov et al., 2012; see also Corbetta, 2005, for a review of the non-human primate literature on posture and manual laterality). Thus, a link between the acquisition of the upright posture and the expression of manual laterality has been documented across development and across species. Our data on infant reaching laterality as a function of walking experience groups are consistent with this scenario. The predicted significant increase in manual laterality was found only in the more experienced walking group, which was the group holding their arms at or below waist level during walking, meaning that they were not relying on their arms so heavily anymore to control balance and moving forward (Ledebt, 2001; Kubo and Ulrich, 2006; Snapp-Childs and Corbetta, 2009). The increase in reaching laterality in that experienced group was also associated with a decrease in EEG coherence, and thus increased regional differentiation in the brain. We could not detect systematic brain asymmetries with our EEG measures (especially to tease apart brain patterns between low lateralized prewalking infants and more lateralized experienced walkers), but future developmental studies should be designed to capture functional brain asymmetries in reaching in toddlers as a function of established hand preference patterns. If we are correct in our assumptions that manual laterality becomes more established after the onset and mastery of upright locomotion, we should discern more distinct lateralized brain response in reaching after experience with upright locomotion compared to developmental periods when upright locomotion has not yet been stably acquired.

Another limitation is that our study was cross-sectional. Because this was a first investigative study of our hypothesis of transfer of learning, we chose to control age in order to be able to compare mapping levels between EEG coherence and behavior as a function walking experience. But cross-sectional approaches only offer a snapshot of unique moments of development, and therefore they are limited when trying to account for the processes that are driving change over time. Because of our design, we cannot infer how the documented changes in EEG coherence can predict or have led to the formation of the new lateralized manual organization found in the experienced walkers. Furthermore, other physiological measures beyond structural EEG coherence should be used to attempt to capture the active brain processes that might be involved in this process of lateralization. In prior work, we argued that preferred hand use develops from a background of repeated fluctuations in behavior, where stabilization and selection of specific patterns of response form as the result of progressive cumulated experiences (Corbetta et al., 2006; Jacobsohn et al., 2014). But to further these issues and better understand how such patterns of lateralization form over time, we need to conduct longitudinal studies that integrate brain and behavioral measures.

Overall, our work confirmed the changing nature of the development of early hand preference, in particular in relation to the development of novel locomotor skills. Our data on the EEG coherence verified and extended Bell and Fox (1996) original findings that each change in motor skills learning is accompanied by changes in brain cortical reorganizations. Over the past decades, neuroscience research has revealed many compelling cases of such brain and behavior mapping and reorganization, but the vast majority of these studies were performed in adults or animals. Here, we show that such mapping across brain and behavioral levels also occur during infancy.

## Conflict of Interest Statement

The authors declare that the research was conducted in the absence of any commercial or financial relationships that could be construed as a potential conflict of interest.
